# Carbetocin Inhibits Behavioral Sensitization to Ethanol in Male and Female Mice, Independent of Corticosterone Levels

**DOI:** 10.3390/toxics11110893

**Published:** 2023-10-31

**Authors:** Beatriz Yamada Costa, Luana Gasparini Santos, Priscila Marianno, Mariana Rae, Marina Gomes de Almeida, Malcon Carneiro de Brito, Rosângela Eichler, Rosana Camarini

**Affiliations:** 1 Department of Pharmacology, Institute of Biomedical Sciences, Universidade de São Paulo, São Paulo 05508-900, Brazil; beatriz.cvs@usp.br (B.Y.C.); primarianno@usp.br (P.M.); marianarae@hotmail.com (M.R.); malconbrito@usp.br (M.C.d.B.); eichler@usp.br (R.E.); 2 School of Pharmaceutical Sciences, Universidade de São Paulo, São Paulo 05508-900, Brazil; luuanagasparini@gmail.com

**Keywords:** behavioral sensitization, ethanol, oxytocin, addiction, estrous cycle, ethanol self-administration

## Abstract

Oxytocin (OXT), a pro-social peptide, is increasingly recognized as a potential protective substance against drug addiction. In the context of ethanol, previous research has shown OXT’s properties in reducing self-administration, alleviating motor impairment in rodents, and reducing craving in humans. However, its role in behavioral sensitization, a neuroadaptive response resulting from repeated drug exposure linked to an increased drug incentive, remains unexplored. OXT is recognized for its role in regulating the hypothalamic–pituitary–adrenal (HPA) axis, in which corticosterone is acknowledged as a significant factor in the development of behavioral sensitization. This study aimed to investigate the effects of carbetocin (CBT), an analogue of OXT, on the expression of behavioral sensitization to ethanol and the concurrent alterations in plasma corticosterone levels in male and female Swiss mice. We also aimed to confirm previous studies on OXT’s impact on ethanol consumption in male mice, but with a focus on CBT, using the two-bottle choice model and the drinking in the dark (DID) methodology. For the sensitization study, the mice received either ethanol (1.8 g/kg, i.p.) or saline treatments daily for 15 consecutive days, followed by treatment with carbetocin (0.64 mg/kg, i.p.) or a vehicle for 6 days. Subsequently, on day 22, all the animals underwent an ethanol challenge to assess the expression of behavioral sensitization. The plasma corticosterone levels were measured on days 21 and 22. The CBT effectively prevented the expression of ethanol-induced behavioral sensitization in both male and female subjects, with no alterations having been detected in their corticosterone levels. In the ethanol consumption study, following an initial phase of ethanol acquisition, the male mice underwent a 6-day treatment with CBT i.p. or saline before being re-exposed to ethanol. We also found a reduction in their ethanol consumption due to the CBT treatment. In conclusion, carbetocin emerges as a promising and effective intervention for mitigating ethanol-induced behavioral sensitization and reducing ethanol intake, highlighting its potential significance in alcohol addiction treatment.

## 1. Introduction

Alcohol, a globally consumed psychoactive substance, has a myriad of adverse consequences for individuals and society [1]. It significantly contributes to the development or exacerbation of over 200 different diseases and health conditions classified in the ICD-10 system [1]. The likelihood of mortality from any of these causes, as well as the risk of developing cancers, escalates as alcohol consumption levels increase, and the level of consumption for minimizing health-related harm is zero [2]. Ethanol consumption is linked to a spectrum of hepatic disorders, encompassing liver inflammation, fatty liver disease, and cirrhosis [3]. Beyond hepatotoxicity, alcohol-derived metabolites contribute to oxidative stress and impaired cognitive function and exert systemic repercussions on multiple organ systems, encompassing the cardiovascular and gastrointestinal systems [4,5].

Alcohol use disorder (AUD) is a health condition that affects both men and women, albeit with notable differences in terms of prevalence, manifestation, and consequences between the genders. The detrimental consumption of alcohol contributes to 7.1% of the global disease burden in males and 2.2% in females [6]. Men exhibit higher rates of alcohol consumption and AUD compared to women [7]. This has often been attributed to cultural and societal factors, including gender-specific expectations that may encourage men to engage in alcohol-related risky behaviors [8]. However, recent studies suggest that the gender gap in AUD is narrowing, with an increasing number of women experiencing alcohol-related issues [9].

Currently, only a few medications are licensed for treating AUD, including disulfiram, naltrexone, acamprosate, and nalmefene. Ongoing research is necessary due to the limitations of the existing drugs for the treatment of AUD [10]. Moreover, tailored treatment approaches that consider gender-specific factors have become increasingly important in addressing AUD. Gender-sensitive interventions, support groups, and healthcare services have been shown to enhance the effectiveness of treatment and recovery strategies for both men and women [11].

One of the emerging treatment options is oxytocin (OXT), a neuropeptide involved in the modulation of different behaviors, such as mood, social interaction, couple formation, and stress [12]. The effect of this neuropeptide on addiction has received great attention [13], with studies indicating that OXT administration decreases alcohol self-administration [14] and reduces cue-reactivity to ethanol in rats and humans [15]. Notably, alcohol-dependent rats exhibited significant changes in their OXT systems, whereas female rats showed no alterations [16]. Furthermore, genetic disruption of the OXT receptor using knockout mice influenced alcohol consumption in female mice, resulting in an increased intake before and after their exposure to stress, while male mice showed no significant genotypic differences [17], indicating sex-specific responses to OXT. 

Although there are several studies demonstrating the effects of OXT on alcohol consumption in both males and females, the role of OXT in the behavioral sensitization to ethanol and potential sex-specific responses, in particular, still remain unknown. While models of self-administration address the rewarding effects of drug abuse [18], behavioral sensitization focuses on neuroadaptive processes, as it is described as the psychomotor manifestation of sensitization in neuronal pathways [19]. Studies using mice have shown that females are more sensitive to ethanol-induced behavioral sensitization than males, pointing to a sex-dependent criteria for this phenomenon [20,21]. 

Although sensitization can be associated with several behaviors, increased locomotor activity is the most commonly studied phenomenon. Nonetheless, sensitization can affect not only behavioral but also neurochemical or neuroendocrine processes, an effect which can be observed in an increase in neurotransmitters’ release or hormonal secretions such as corticosterone [22], for instance. Corticosterone plays an important role in the development of behavioral sensitization, since the activation of the hypothalamic–pituitary–adrenal (HPA) axis has been described to increase drug use [23]. Specifically, regarding behavioral sensitization to alcohol, a cross-sensitization between alcohol and stress has also been reported [24,25]. In this context, OXT contributes to the regulation of stress responses through its interaction with the HPA axis [26]. This interaction involves a cascade of neuroendocrine processes wherein OXT may influence the release of hormones, including glucocorticoids [27]. 

In our research, we chose to investigate the effects of carbetocin (CBT), a synthetic analogue of OXT. Despite its structural similarity to OXT, it exhibits a longer half-life. CBT has been investigated for its potential to prevent the priming-induced reinstatement of morphine-seeking behaviors [28,29] and has the advantage of not inducing any alterations in the plasma corticosterone levels [28]. In this study, we aimed to assess the impact of CBT on the expression of behavioral sensitization to ethanol and its effects on plasma corticosterone levels in male and female mice. Additionally, we investigated the influence of CBT on ethanol intake in male mice.

## 2. Materials and Methods

### 2.1. Animals 

Thirty-two male and thirty-two female Swiss mice were housed in groups of four, with food and water ad libitum, in an experimental room, with controlled temperature (24 ± 2 °C) and light conditions (light/dark cycle of 12 h; lights on at 7:00 a.m.). Swiss mice were used owing to their sensitivity to the stimulant effects of ethanol, making them useful for studying behavioral sensitization in male and female mice [30,31]. 

For the ethanol consumption experiment, twenty-eight adult male C57BL/6 mice, 8–10 weeks old, were housed in groups of four, with food and water ad libitum, with controlled temperature and light conditions (light/dark cycle of 12 h; lights off at 7:00 a.m.). C57BL/6 mice were chosen for their genetic predisposition to voluntarily consume significant amounts of ethanol, but their low sensitivity to behavioral sensitization, making them a good choice for alcohol intake studies [32,33,34]. The animals were acclimatized to the reverse cycle at least 2 weeks before the experiments. Red incandescent lights were utilized during the dark phase to facilitate mice handling by the investigators. 

All the procedures were approved by the Ethics Committee on the Use of Animals of the Institute of Biomedical Sciences (University of Sao Paulo) (CEUA—ICB/USP), under CEUA numbers 9998280518 and 4512140222 and protocol 25/2016, in accordance with Law 11,794 of 8 October 2008, Decree 6899 of 15 July 2009, as well as with the rules issued by the National Council for Control of Animal Experimentation (CONCEA). Efforts were made to minimize pain and suffering and reduce the use of animals. Two male Swiss mice were excluded from this cohort due to their aggressive behavior and two male C57BL/6 mice died from unknown causes.

### 2.2. Drugs 

Ethanol (95%; Labsynth, Diadema, SP, Brazil) was administered intraperitoneally (i.p.) in a 20% (*v*/*v*) solution, prepared with a saline solution (NaCl 0.9%), at a dose of 1.8 g/kg. The saline solution was used as a control solution and injected i.p. For the voluntary ethanol intake, ethanol was diluted to 20% (*v*/*v*) in tap water. Carbetocin (CBT) (Sigma-Aldrich, St. Louis, MO, USA), a synthetic analog of OXT, was dissolved in a saline solution and administered i.p. for 6 days, following the locomotor sensitization protocol, and, before the re-exposure to ethanol in the DID paradigm = for 6 consecutive days, at a dose of 6.4 mg/kg. CBT was chosen due to its longer half-life (85–100 min) compared to OXT (3–5 min) and its stability, facilitating its handling for a longer period of time [35]. 

### 2.3. Identification of the Estrous Cycle Phase 

In female mice, the phases of the estrous cycle were identified with a fresh cytological analysis of vaginal lavage. The animals were properly restrained, and a careful vaginal wash was performed with 15 µL of 0.9% saline solution. The saline was injected, aspirated, and then placed on a histological slide for microscope viewing and phase identification [36]. This procedure ensured a consistent timing for blood collection and measurement of corticosterone.

### 2.4. Experimental Design

#### 2.4.1. Effects of Carbetocin on Ethanol-Induced Behavioral Sensitization

**Figure 1:**
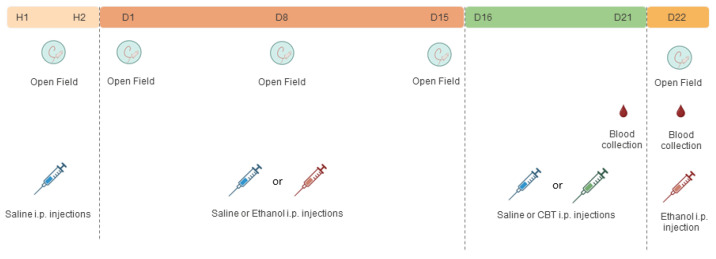
Experimental design 1: The experimental design involved the initial administration of saline in H1 and H2 (habituation days), followed by a subsequent treatment with either saline or 1.8 g/kg of ethanol (from days D1 to D15). From days D16 to D21, the mice received injections of either saline or 6.4 g/kg of carbetocin (CBT). On day 22 (D22), all the mice were challenged with 1.8 g/kg of ethanol, resulting in the following four groups: SAL-SAL, SAL-CBT, ETOH-SAL, and ETOH-CBT.

The experimental design is depicted in Figure 1. The locomotor activity was evaluated in an open field, a plexiglass arena measuring 40 cm in diameter, and a wall measuring 50 cm in height. The animals received the saline or ethanol injections according to the experimental group and, after five minutes, were placed into the center of the apparatus. The total horizontal locomotor activity was evaluated for a period of 5 min, as the peak in the locomotor activation induced by ethanol occurs between 5 and 10 min after ethanol administration [37,38]. All the experiments were performed between 9:00 a.m. and 10:00 a.m. The activity was recorded using a digital camera and a video-capture system. The EthoVision^®^ software version 11.5.1026 (Noldus, The Netherlands) was used to quantify the distance covered by each animal, as previously described [39].

For the first two days (H1 and H2), all the animals received an intraperitoneal saline solution and had their locomotor activity evaluated in the open field, to familiarize them with the experimenter’s handling and minimize the novelty effect of the apparatus. 

Following the habituation period, the mice were randomly assigned to either the saline (SAL) or 1.8 g/kg ethanol (EtOH) groups. During the 15 days of treatment (D1–D15), the mice received daily injections of either SAL or 1.8 g/kg of ETOH i.p., based on their group assignment. From D16 to D21, the animals underwent a period of ethanol withdrawal, during which half of the animals in each group received 6.4 mg/kg of CBT i.p. and the other half received isovolumetric injections of saline as a control. On D22, all the animals were challenged with an injection of 1.8 g/kg of ethanol. For each sex, we established four distinct groups, as follows: SAL-SAL, SAL-CBT, ETOH-SAL, and ETOH-CBT (n = 7–8/group).

The locomotor activity was evaluated on days H1, H2, D1, D8, D15, and D22. In addition, blood samples from the caudal vein were collected for the subsequent measurement of plasma corticosterone on D21, between 1:00 p.m. and 3:00 p.m., and D22, after the behavioral test, for the subsequent corticosterone and ethanol measurements. 

#### 2.4.2. Effects of Carbetocin on Ethanol Consumption

**Figure 2:**
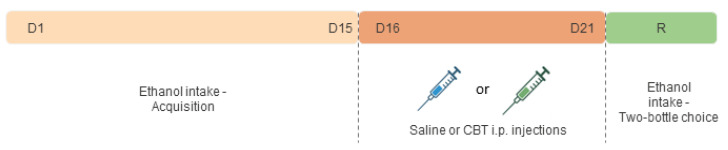
Experimental design 2. After an initial 15-day period of alcohol acquisition (D1–D15) using the DID paradigm, the mice underwent a 6-day treatment phase with either saline or 6.4 mg/kg of carbetocin (CBT) i.p (from days D16 to D21). The administration of the CBT occurred at different time points, either 1 h or 24 h prior to re-exposure (R). The R phase involved the re-exposure to the two-bottle choice test (water vs. ethanol). Three groups were formed, as follows: SAL, CBT-1H, and CBT-24H.

The experimental design is shown in Figure 2. We employed the drinking in the dark (DID) protocol, using the two-bottle choice method, to assess the effects of CBT on ethanol consumption. Three hours after the onset of the dark phase, the animals were single-housed with free access to two bottles: one contained ethanol (95% *v*/*v*; Labsynth, SP, Brazil) diluted to 20% (*v*/*v*) in tap water, and the other contained tap water. The mice were allowed to freely consume both solutions for a 2 h period. Subsequently, the bottles were removed, and the mice were returned to their respective home cages. The bottles were weighed both before and immediately after the consumption sessions, and the differences in weight were converted into the volumes of ethanol and water solutions consumed. The ethanol consumption in grams per kilogram (g/kg) was determined by taking into account the density of the ethanol, the concentration of the solution, the quantity of the solution consumed, and the body weight of each subject. The solutions were replaced daily, and the positions of the bottles were regularly interchanged to eliminate potential side preferences. Throughout the DID procedure, a separate cage with two bottles was employed as a control to account for any liquid loss from handling or evaporation. The volume lost in these control bottles was subtracted from the measured volume of ethanol or water consumed by each animal.

Since previous studies showed no OXT-specific changes in female mice after ethanol exposure [16], the DID protocol was employed to evaluate voluntary ethanol consumption only in male mice, with modifications adapted from a previous study [40,41]. With this study we sought to investigate whether CBT would yield results consistent with previous research conducted with OXT. This investigation involved an experimental paradigm comprising distinct phases, including an initial period of acquisition, followed by a withdrawal phase, and, ultimately, re-exposure to ethanol. The mice were exposed to the DID paradigm for 15 days to ensure the stabilization of their ethanol consumption (acquisition phase). Following the acquisition phase, the animals were randomly distributed into three groups—CTL (n = eight), CBT-1H (n = nine), and CBT-24H (n = nine)—and treated accordingly, with either saline or CBT (6.4 mg/kg), for six consecutive days during a period of ethanol deprivation. The CBT was administered at two different time points: either 1 h (CBT-1H) or 24 h (CBT-24H) prior to re-exposure (R). Subsequently, the animals were given two bottles, with free access to ethanol (20%) and water for 24 h, and their consumption was measured at both the 2 h and 24 h marks from the onset of drinking, following the protocol described in Marianno et al., 2017 [41].

### 2.5. Blood Collection for Biochemical Analysis

Blood collection for a subsequent corticosterone measurement was taken on D21, between 1:00 p.m. and 3:00 p.m. Approximately 100 μL of blood was collected from the caudal vein and placed in microcentrifuge tubes containing heparin (100 U/mL, in the volume of 10% of the total volume of the blood collected). The samples (n = 7/group) were centrifuged at 2000× *g* at 4 °C for 10 min and the plasma was transferred to a clean tube and stored at −80 °C. The corticosterone levels were determined using the IBL Corticosterone Enzyme Immunoassay Kit (Tecan Trading AG, Männedorf, Switzerland), following the manufacturer’s procedures. 

Blood samples were collected on D22, after the animals were euthanized, for a subsequent corticosterone and ethanol measurement, between 9:00 a.m. and 11:00 a.m. Approximately 250 μL of blood were placed in microcentrifuge tubes containing heparin (250 U/mL, in the volume of 10% of the total volume of the blood collected). The samples were processed as described above. The corticosterone levels (n = 5–7/group) and the blood ethanol concentration (BEC) (n = 5/group) were assayed. The BEC was analyzed using the Ethanol Assay Kit Abcam (Abcam plc, Cambridge, UK), following the manufacturer’s procedures. Some of the samples underwent hemolysis, resulting in a reduction in the number of samples.

### 2.6. Statistical Analysis

The results were submitted for statistical analysis using the Statistica program, version 7.0. Levene’s test was employed to assess the homogeneity of variances. A four-way analysis of variance (ANOVA) for repeated measures was performed to analyze the data related to the mice’s locomotor activity in the behavioral sensitization experiment, with “pretreatment” (SAL or ETOH), “treatment” (SAL or CBT), and “sex” (MALE or FEMALE) as the between-group statistical factors, and “time” as the repeated measure. Follow-up three-way ANOVAs for repeated measures were performed for each sex. The locomotor response to the ethanol challenge (D22) was analyzed with two-way ANOVAs, using “pretreatment” and “treatment” as the between-group factors. For the analysis of the corticosterone levels, a three-way ANOVA (pretreatment X treatment X sex) was followed up with two-way ANOVAs using “pretreatment” and “treatment” as the between-group factors for males. For females, we included the diestrus vs. non-diestrus phases as a factor to control for hormonal variations (pretreatment X treatment X estrous phase). For the analysis of the blood ethanol concentration, we used a three-way ANOVA (pretreatment X treatment X sex). 

As for the data from the mice’s ethanol intake during the acquisition phase, we conducted a one-way ANOVA for repeated measures, with “time” as the repeated measure. The analysis of the re-exposure (R) data (2 h) was conducted using a two-way ANOVA for repeated measures, considering time as the repeated measure (with two levels: mean of the last 5 days of acquisition and re-exposure) and group (CTL, CBT-1H, CBT-24H) as the between-subjects factor. The analysis of re-exposure to ethanol for 24 h was performed using a one-way ANOVA. A Newman–Keuls post hoc test was used to compare the means when statistical significance was found in the repeated measures, and, for the non-repeated measures, the Tukey test was used. The values of *p* < 0.05 were considered significant. Statistical details other than those explicitly mentioned in the main text can be found in the Supplementary Materials. 

## 3. Results

### 3.1. CBT Inhibited the Expression of Behavioral Sensitization in Male and Female Mice

**Figure 3:**
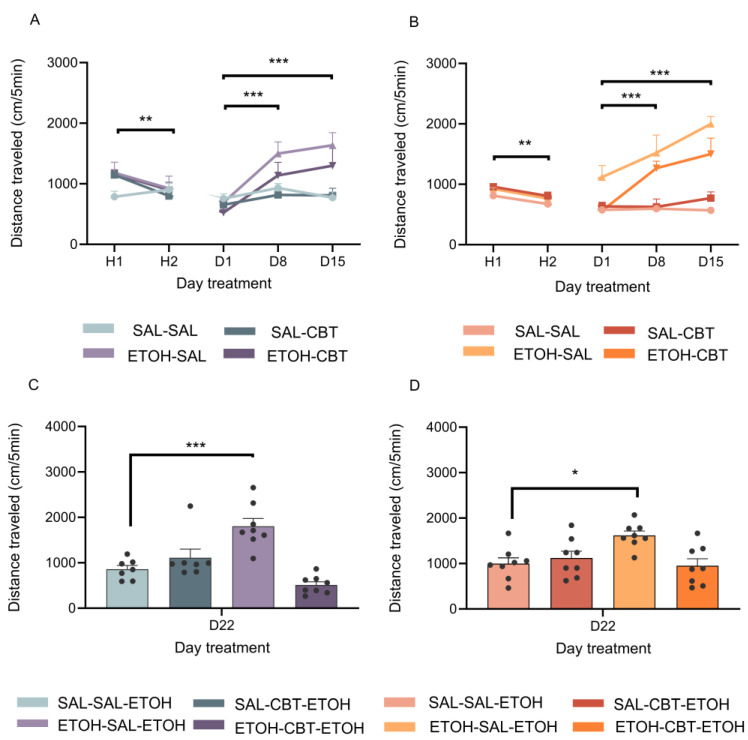
Effects of CBT on the expression of locomotor sensitization. The figure displays locomotor activity (cm) measured over 5 min time-periods. After 2 days of saline injections (H1 and H2), the mice received either SAL or ETOH i.p. injections daily for 15 days (D1–D15) based on their group assignment [Figures (**A**) (male) and (**B**) (female)]. From days D16 to D21, the animals underwent a period of ethanol withdrawal, during which half of the animals in each group received 6.4 mg/kg of CBT i.p. and the other half received injections of saline as a control. On the following day (D22) [Figures (**C**) (male) and (**D**) (female)], all the animals were challenged with an injection of 1.8 g/kg of ethanol. For each sex, four distinct groups were established, as follows: SAL-SAL (n = 7–8/group), SAL-CBT (n = 7–8/group), EtOH-SAL (n = 8/group), and EtOH-CBT (n = 8/group). The locomotor activity in H2 was lower than in H1. The locomotor activity of the mice subjected to ethanol treatment was higher on D8 and D15 compared to D1 in both the male and female mice. The activity of the ETOH-SAL-ETOH group on D22 differed from that of the SAL-SAL-ETOH; * *p* < 0.05, ** *p* < 0.01, *** *p* < 0.001. The data represent the mean ± SEM. Figure 3C,D feature individual data points.

A four-way ANOVA used to analyze the locomotor activity on H1 and H2 (Figure 3A,B) revealed significant effects of sex [F(1,54) = 5.28, *p* < 0.05] and time [F(1,54) = 18.57, *p* < 0.001]. The female mice exhibited a reduced motor activity compared to the males, which may be attributed to differences in sensitivity to novelty. The decrease in locomotor activity from H2 to H1 indicates a typical habituation response to the apparatus. Following that, we performed a four-way ANOVA to analyze the locomotor activity on D1, D8, and D15. Given the absence of significant sex differences [F(1,54) = 0.06, *p* = 0.80], we continued the analysis with separate three-way ANOVAs for each sex. 

The statistical analysis of the locomotor activity in the male mice on D1, D8, and D15 revealed significant effects of the pretreatment [F(1,26) = 8.81, *p* < 0.01] and of the time [F(2,52) = 16.22, *p* < 0.001], as well as an interaction between the pretreatment and the time [F(2,52) = 9.22, *p* < 0.01]. As depicted in Figure 3A, the locomotor activity of the male mice who had been subjected to ethanol treatment was higher on D8 and D15 compared to D1, as detected with the post hoc Newman–Keuls test. These results suggest that the male mice exhibited behavioral sensitization starting from D8, which was further confirmed by their increased locomotor activity on D15. 

Similar results were observed in the female mice (Figure 3B). A three-way ANOVA for repeated measures also detected significant effects of the pretreatment [F(1,28) = 63.70, *p* < 0.001] and of the time [F(2,56) = 11.20, *p* < 0.001], as well as an interaction between the pretreatment and the treatment [F(1,28) = 9.22, *p* < 0.01] and an interaction between the pretreatment and the time [F(2,56) = 8.56, *p* < 0.001]. The post hoc Newman–Keuls test showed significant differences on D8 and D15 compared to D1 in the ethanol-pretreated group. 

Figure 3C shows the locomotor activity of the male mice measured on D22, when all the mice received a challenge injection of 1.8 g/kg of ethanol. A two-way ANOVA revealed significant effects of the treatment [F(1,26) = 13.94, *p* < 0.001] and an interaction between the pretreatment and the treatment [F(1,26) = 30.86, *p* < 0.001]. The animals previously exposed to repeated ethanol treatment followed by saline during the abstinence period (ETOH-SAL-ETOH) exhibited a heightened locomotor activity compared to the animals who had been pre-exposed to saline and were administered saline during this phase (SAL-SAL-ETOH), indicating a more pronounced response in the mice subjected to repeated ethanol administration as opposed to those receiving a single, acute ethanol injection. These data confirmed the expression of ethanol-induced behavioral sensitization in the ETOH-SAL group. No significant difference was detected in the locomotor activity of the ETOH-CBT-ETOH group compared to that of the SAL-SAL-ETOH group, suggesting that CBT was effective in reversing ethanol sensitization. No significant differences were found between the SAL-SAL-ETOH and SAL-CBT-ETOH groups, showing that CBT did not affect the locomotion of these animals. 

Likewise, a two-way ANOVA applied to the data from the female mice on D22 revealed a significant treatment effect [F(1,28) = 4.12, *p* = 0.05] and an interaction between the pretreatment and the treatment [F(1,28) = 8.77, *p* < 0.01]. The post hoc test indicated that the ETOH-SAL-ETOH group exhibited a greater locomotor activity than the SAL-SAL-ETOH (Figure 3D) group. No significant differences were found in the locomotor activity between the ETOH-CBT-ETOH group and the SAL-SAL-ETOH or SAL-CBT-ETOH groups. The results confirm the efficacy of CBT in reversing ethanol sensitization in the female mice as well. In alignment with the previous results found in the male mice, no significant differences were found between the SAL-SAL-ETOH and SAL-CBT-ETOH groups, underscoring that CBT did not exert an impact on the locomotion of these animals. 

### 3.2. CBT Influence on Behavioral Sensitization Is Not Mediated by Alterations in the Stress Hormone Corticosterone

**Figure 4:**
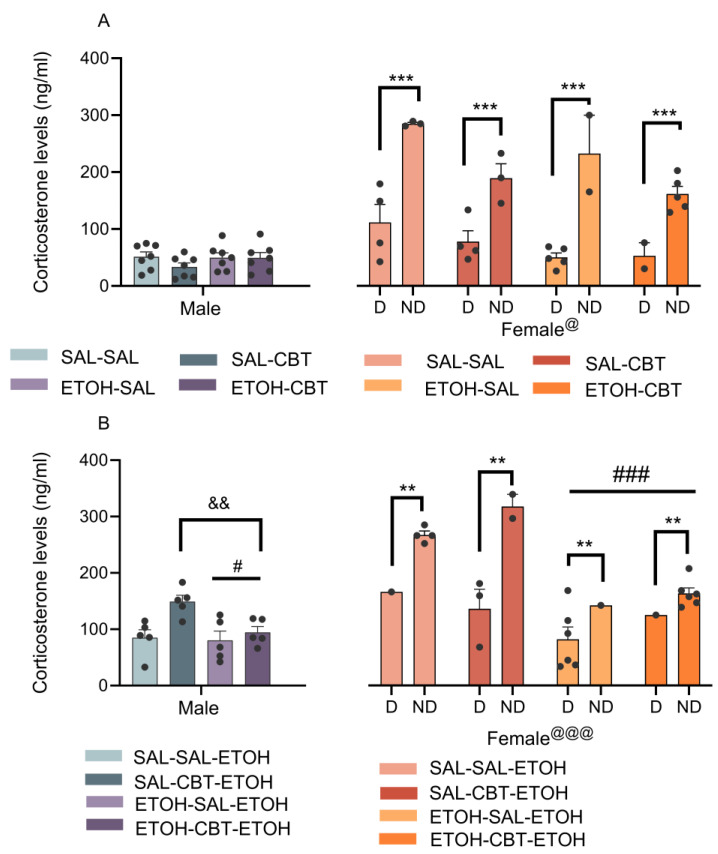
Effects of carbetocin (CBT) and ethanol on plasma corticosterone levels. The male and female animals were pretreated with either saline or ethanol for 15 days, followed by treatment with either saline or CBT during a 6-day abstinence period, and they were challenged with 1.8 g/kg of ethanol on the following day. The corticosterone concentrations were measured on D21 (n = 7/group, Figure (**A**)) and D22 (n = 5–7/group, Figure (**B**)). D: diestrus; ND: non-diestrus. @ *p* < 0.05 and @@@ *p* < 0.001: the female mice showed higher corticosterone levels compared to the male mice. # *p* < 0.05 and ### *p* < 0.001: the mice pretreated with ethanol (ETOH-) exhibited lower levels of corticosterone than those pretreated with saline (SAL-). && *p* < 0.01: the mice treated with CBT (SAL-CBT and ETOH-CBT) displayed higher corticosterone levels than those treated with saline (SAL-SAL and ETOH-SAL). ** *p* < 0.01 and *** *p* < 0.001: the corticosterone levels in the non-diestrus are higher than in the diestrus. The data represent the mean ± SEM. Figure 4A,B feature individual data points.

The analysis of the plasma corticosterone levels from D21 (Figure 4A) with a three-way ANOVA revealed a significant effect associated with the sex factor [F(1,48) = 5.92, *p* < 0.05], with the female mice exhibiting higher corticosterone levels than the male mice. No other effects or interactions were found. A follow-up analysis of the data from the male mice using a two-way ANOVA confirmed no significant main effects or interactions. For the female mice, we considered the diestrus and non-diestrus phases as a variable (cycle). In this case, we found an effect of the cycle [F(1,20) = 73.51, *p* < 0.001]. While ANOVAs unveiled effects related to the pretreatment [F (1,20) = 6.16, *p* = 0.02] and the treatment [F (1,20) = 8.64, *p* = 0.01], interpreting these findings is challenging due to the influence of hormonal variations and the unequal distribution of female mice across different phases within each group. A post hoc analysis revealed higher corticosterone levels in the non-diestrus phases compared to the diestrus.

The analysis of the plasma corticosterone levels from D22 (Figure 4B) using a three-way ANOVA revealed significant effects of the sex [F(1,36) = 22.27, *p* < 0.01] and pretreatment [F(1,36) = 18.72, *p* < 0.001] factors, as well as interactions between the sex and the pretreatment [F(1,36) = 5.68, *p* < 0.05], and among the sex, the pretreatment, and the treatment [F(1,36) = 6.38, *p* < 0.05]. No other main effects or significant interactions were observed. A post hoc analysis revealed higher corticosterone levels in the female mice compared to the male mice, as observed in D21. 

Subsequently, we conducted a two-way ANOVA to analyze the data from the male mice, revealing a significant effect of the pretreatment [F(1,16) = 5.02, *p* < 0.05]. The mice who had been previously exposed to ethanol exhibited lower corticosterone levels compared to the ethanol-naïve mice when both groups were challenged with ethanol. Additionally, a significant treatment effect [F(1,16) = 8.66, *p* < 0.05] demonstrated that CBT increased the corticosterone levels in the male mice, irrespective of the pretreatment. 

The analysis of the corticosterone levels in the female mice using a 3-way ANOVA revealed an effect of the estrous cycle [F (1,16) = 12.26, *p* < 0.01], indicating higher hormone levels during the non-diestrus phases compared to the diestrus. We also found an effect of the pretreatment, similar to that observed in the male mice [F(1,16) = 22.49, *p* < 0.01]. However, differently from the male mice, CBT treatment did not alter the corticosterone levels in the female mice on D22, when they were challenged with ethanol [F(1,16) = 0.12, *p* = 0.73]. 

### 3.3. CBT Does Not Alter Ethanol Metabolism 

**Figure 5:**
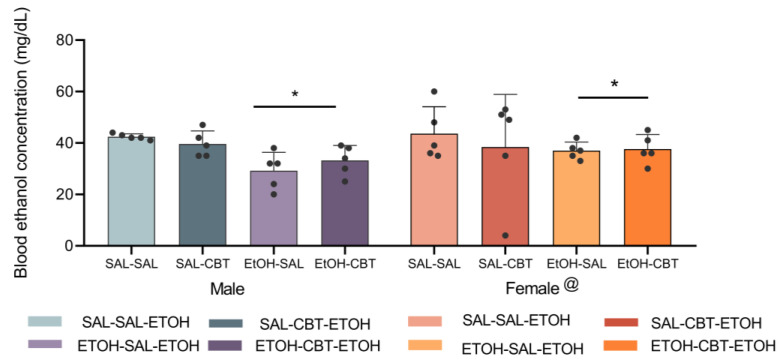
Effects of CBT and ethanol on blood ethanol concentration (BEC). The male and female animals were pretreated with either saline or ethanol for 15 days (D1 to D15), followed by treatment with either saline or CBT during a 6-day abstinence period (D16 to D21). On day 22, all the animals received an injection of 1.8 g/kg of ethanol. The BEC was measured on D22 (n = 5/group). @ *p* < 0.05: the female mice showed higher BECs compared to the male mice. * *p* < 0.05: the ethanol pretreatment resulted in a lower BEC compared to the saline pretreatment. Figure 5 features individual data points.

The analysis of the blood ethanol concentration (BEC) (Figure 5) using a three-way ANOVA revealed statistically significant effects related to the sex [F(1,32) = 5.77, *p* < 0.05] and the pretreatment [F(1,32) = 17.95, *p* < 0.001], but it did not show any statistically significant differences associated with the treatment [F(1,32) = 0.22, *p* = 0.64]. The female mice showed higher BECs compared to the male mice. Moreover, the mice with prior ethanol exposure exhibited a lower BEC compared to the alcohol-naïve mice. No other main effects or interactions were observed. 

### 3.4. CBT Decreases Ethanol Intake in Male Mice

**Figure 6:**
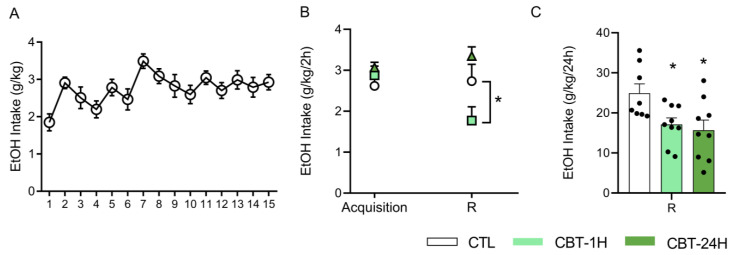
CBT decreases ethanol intake (g/kg) in male mice. The male mice were exposed to the DID paradigm for 15 days to ensure the stabilization of ethanol consumption (acquisition phase) (**A**). Following the acquisition phase, the animals were randomly distributed into three groups—control (CTL, n = eight), CBT-1H (n = nine), and CBT-24H (n = nine)—and treated accordingly for 6 consecutive days. After a six-day period of ethanol deprivation, the mice were re-exposed to the two-bottle choice (R) method, with free access to ethanol and water for 24 h. The consumption was measured at both 2 (**B**) and 24 h (**C**) from the initiation of drinking. * *p* < 0.05: differs from CTL. DID = drinking in the dark. Figure 6C features individual data points.

The analysis of the data from the last 5 days of the 15-day acquisition phase using a one-way ANOVA for repeated measures revealed no differences among them (F(4,180) = 0.56, *p* = 0.69; Figure 6A). This indicates that, at the end of the acquisition phase, the mice had reached stable levels of ethanol consumption.

Figure 6B shows the ethanol intake averaged over the final 5 days of the acquisition phase and the consumption after 2 h from re-exposure. The ANOVA revealed significant effects of the treatment (F(2,23) = 4.11, *p* < 0.05) and a treatment X time interaction (F(2,23) = 5.14, *p* < 0.05), with no significant effect of the time (F(2,23) = 1.45, *p* = 0.24). During the 2 h re-exposure period, the CBT-1H group exhibited a significant reduction in their ethanol intake compared to both the acquisition phase and the other two groups. 

Ethanol consumption was additionally measured at the end of the 24 h period of free access to both ethanol and water, during the re-exposure phase (Figure 6C). The one-way ANOVA revealed a significant treatment effect (F(2,23) = 4.90, *p* < 0.05), with the groups that received CBT exhibiting a reduction in their ethanol consumption compared to the control group.

## 4. Discussion

To our knowledge, this study is the first to demonstrate that CBT can reverse ethanol-induced behavioral sensitization in both male and female Swiss mice. CBT treatment alone failed to induce changes in the mice’s locomotor activity; hence, it can be inferred that the reduction in behavioral sensitization is not contingent upon alterations in locomotor activity induced by CBT.

The concept of sensitization, initially described by Segal and Mandell in 1974 [42], entails the gradual and persistent amplification of specific behaviors after repeated exposure to stimulant drugs. It serves as a well-studied model of neuroplasticity. Following intermittent stimulant drug treatment, such as amphetamine or cocaine, or after administering stimulant ethanol doses, sensitized behaviors may manifest with increased intensity, faster onset, or at lower doses than before sensitization [43,44]. There is strong evidence linking behavioral sensitization to changes in limbic neurochemical systems, which play a role in various psychiatric and substance use disorders [45]. 

In a recent study in our laboratory [46], we observed that CBT enhanced the rewarding effects of ethanol, as measured with conditioned place preference (CPP), which primarily reflects the rewarding aspects of a substance and relies on Pavlovian learning mechanisms [47]. In the present study, we adopted the behavioral sensitization paradigm to model a different facet of addiction, aligned with the incentive-sensitization theory proposed by Robinson and Berridge in 1993 [48]. Our findings suggest that, although CBT may enhance the rewarding effect of ethanol in certain contexts, it can also exert protective effects against the expression of ethanol-induced behavioral sensitization. These results highlight the complex role of CBT in modulating different aspects of ethanol addiction. It is important to emphasize that, while sensitization may contribute to the development of addiction by enhancing the incentive salience of drugs, it does not imply an inescapable cycle of substance use. One can experience sensitization without progressing to a chronic substance dependence, and, likewise, individuals can develop substance use disorders without exhibiting sensitization. 

Regarding our findings about corticosterone levels, CBT did not change corticosterone levels on D21, in agreement with previous studies [28]. On D22, following a challenge injection with ethanol, the levels of this hormone decreased in the mice who had been previously exposed to ethanol compared to those who had been pre-exposed to saline. This reduction in corticosterone levels in the mice with prior ethanol exposure, as opposed to those receiving an acute ethanol dose, suggests a potential development of tolerance or adaptation to the stress-inducing effects of ethanol. While CBT treatment increased the corticosterone levels in the male mice, the female mice did not exhibit the same response, indicating that the results may be affected by estrous cycle variations. Our study did not reveal any specific effect of CBT on the corticosterone levels of the male or female mice who had undergone ethanol sensitization. This suggests that the reversal of sensitization using CBT is not contingent upon corticosterone levels and involves a distinct pathway or mechanism for modulating sensitization. The OXT system contributes to the regulation of stress responses through its interaction with the HPA axis [49], but its potential to alleviate addiction-related behaviors exacerbated by stress has yet to be explored [50]. When it comes to intranasal OXT administration in individuals with AUD, for example, its effects on alcohol craving appear to vary, influenced by the individuals’ anxiety levels [51]. 

The higher levels of corticosterone in the female mice compared to the male mice can likely be attributed to hormonal variations, given that approximately 50% of the female mice were in non-diestrus phases. When we analyzed the corticosterone levels considering the estrous cycle, the non-diestrus phases differed from the diestrus, exhibiting higher levels of corticosterone. These findings underscore the significance of identifying specific phases of the estrous cycle, considering the associated hormonal variations. Diestrus, known for its longer duration, is characterized by a period of quiescence and lower estradiol levels [51,52,53]. In contrast, the pre-ovulatory period, considered as non-diestrus, is characterized by an increased estradiol secretion [54]. Glucocorticoids and estradiol can mutually influence each other [55], as evidenced by studies demonstrating the enhancement of corticosterone secretion with estradiol administration in female rats [56]. The observed interplay between estradiol and corticosterone highlights the complexity of hormonal regulation during different phases of the estrous cycle. 

While we observed no sex-specific differences in ethanol sensitization or the influence of CBT treatment on this response, previous research by Hansson et al. (2018) [15] revealed significant alterations in the OXT system among male, dependent rats and in the post-mortem brains of human individuals with alcohol addiction, but not in the female subjects [16]. The divergent responses in the OXT system between male and female subjects highlight the need for gender-specific considerations in addiction research and treatment approaches.

This study also revealed that the female mice exhibited higher BEC levels compared to the male mice, likely attributed to differences in metabolism, body composition, and alcohol absorption rates between the sexes. In fact, in humans, men typically exhibit higher gastric alcohol dehydrogenase (ADH) activity compared to women, leading to a lower, peak BEC in men compared to women [42]. Similar results have been described for mice [43]. It is also worth noting that the mice with prior ethanol exposure exhibited a lower BEC compared to the alcohol-naïve mice, as previously demonstrated. This phenomenon is likely attributable to the development of pharmacokinetic tolerance. Of greater significance, the treatment with CBT did not result in any changes in the BEC levels, suggesting that the CBT mechanism for reducing ethanol sensitization is unlikely to be linked to alterations in ethanol metabolism. 

By using CBT, our research substantiated prior findings seen in studies employing OXT which demonstrated its effectiveness in reducing cue-induced reinstatement response in male, dependent rats [15] and ethanol consumption in various self-administration models in male mice [14]. More recently, King et al. (2021) [57] demonstrated the involvement of endogenous OXT in the hypothalamus in controlling ethanol consumption and suggested that signaling through OXT receptors plays a role in reducing ethanol consumption in a binge-like drinking model. In this study, we further elucidated the effects of CBT administration both 1 h and 24 h before ethanol consumption in a two-bottle choice model. Our results revealed that CBT effectively reduced ethanol intake when the bottles were available for the 24 h session. However, in the 2 h session, only the CBT injection administered 1 h prior to the session demonstrated a significant decrease in ethanol intake. It is important to consider the context of ethanol withdrawal effects on anxiety. 

The observed reduction in ethanol intake might indicate that CBT has the potential to alleviate anxiety or craving associated with ethanol withdrawal, contributing to a decrease in consumption. In fact, OXT has been shown to modulate stress, anxiety, and craving behaviors (see Rae et al. 2022 [58] as review). The fact that CBT effectively reduced ethanol intake during the 24 h session, regardless of whether it was administered 1 h or 24 h before the session, suggests that CBT appears to have a sustained efficacy in reducing ethanol intake during prolonged access to ethanol, irrespective of the timing of its administration.

It is important to highlight that the effects of OXT have been tested on other drugs, such as methamphetamine, and that the results are promising, showing a dose-dependent attenuation of motor hyperactivity through OXT administration, an effect blocked by an OXT antagonist [59]. Furthermore, with regard to opioids, OXT has been shown to decrease the acquisition and maintenance of heroin self-administration [60].

Behavioral sensitization to ethanol can also be accompanied by changes in other behaviors, such as reactivity to stress, reward sensitivity, and cognitive function. More research is needed to assess the effects of CBT on other aspects of behavioral sensitization to ethanol. 

We can conclude that CBT attenuates the neuroplastic events underlying behavioral sensitization in male and female mice and decreases ethanol intake in male mice. It should be emphasized that the relationship between behavioral sensitization and dependence is still debatable. Although there are studies showing that sensitized animals are more vulnerable to increased ethanol consumption [61], this agreement is not unanimous [62]. Nevertheless, the role of sensitization in the neuroadaptive processes that occur with repeated drug exposure should be considered as a phenomenon related to the psychological desire or “wanting” for the drug [48]. These findings point to CBT as a potential therapeutic tool for addressing alcohol use disorders and reducing the health-toxic risks associated with excessive ethanol consumption.

## Data Availability

The data presented in this study are available on request from the corresponding author.

## Supplementary Materials

The following supporting information can be downloaded at https://www.mdpi.com/article/10.3390/toxics11110893/s1: Table S1: Statistical analysis.

## References

[1] World Health Organization (2018). Global Status Report on Alcohol and Health 2018.

[2] GBD 2016 Alcohol and Drug Use Collaborators (2018). The global burden of disease attributable to alcohol and drug use in 195 countries and territories, 1990–2016: A systematic analysis for the Global Burden of Disease Study 2016. Lancet Psychiatry.

[3] Osna N.A., Donohue T.M., Kharbanda K.K. (2017). Alcoholic Liver Disease: Pathogenesis and Current Management. Alcohol. Res..

[4] El-Mas M.M., Abdel-Rahman A.A. (2019). Role of Alcohol Oxidative Metabolism in Its Cardiovascular and Autonomic Effects. Adv. Exp. Med. Biol..

[5] Vijayraghavan S., Porcher L., Mieczkowski P.A., Saini N. (2022). Acetaldehyde makes a distinct mutation signature in single-stranded DNA. Nucleic Acids Res..

[6] World Health Organization (2023). World Health Statistics 2023: Monitoring Health for the SDGs, Sustainable Development Goals.

[7] Goh C.M.J., Asharani P.V., Abdin E., Shahwan S., Zhang Y., Sambasivam R., Vaingankar J.A., Ma S., Chong A.S., Subramaniam M. (2022). Gender Differences in Alcohol Use: A Nationwide Study in a Multiethnic Population. Int. J. Ment. Health Addict..

[8] Sudhinaraset M., Wigglesworth C., Takeuchi D.T. (2016). Social and Cultural Contexts of Alcohol Use: Influences in a Social-Ecological Framework. Alcohol. Res..

[9] White A.M. (2020). Gender Differences in the Epidemiology of Alcohol Use and Related Harms in the United States. Alcohol. Res..

[10] Camarini R. (2019). Mesenchymal stem cells as new perspective for the treatment of alcohol use disorder. Gene Ther..

[11] Green C.A. (2006). Gender and use of substance abuse treatment services. Alcohol. Res. Health.

[12] Gimpl G., Fahrenholz F. (2001). The oxytocin receptor system: Structure, function, and regulation. Physiol. Rev..

[13] Ryabinin A.E., Fulenwider H.D. (2021). Alcohol and oxytocin: Scrutinizing the relationship. Neurosci. Biobehav. Rev..

[14] King C.E., Griffin W.C., Luderman L.N., Kates M.M., McGinty J.F., Becker H.C. (2017). Oxytocin reduces ethanol self-administration in mice. Alcohol. Clin. Exp. Res..

[15] Hansson A.C., Koopmann A., Uhrig S., Bühler S., Domi E., Kiessling E., Ciccocioppo R., Froemke R.C., Grinevich V., Kiefer F. (2018). Oxytocin Reduces Alcohol Cue-Reactivity in Alcohol-Dependent Rats and Humans. Neuropsychopharmacology.

[16] Hansson A.C., Spanagel R. (2021). No changes in the oxytocin system in alcohol-dependent female rodents and humans: Towards a sex-specific psychopharmacology in alcoholism. Addict. Biol..

[17] Rodriguez K.M., Smith B.L., Caldwell H.K. (2020). Voluntary alcohol consumption is increased in female, but not male, oxytocin receptor knockout mice. Brain Behav..

[18] Bardo M.T., Bevins R.A. (2000). Conditioned place preference: What does it add to our preclinical understanding of drug reward?. Psychopharmacology.

[19] Carrara-Nascimento P.F., Hoffmann L.B., Flório J.C., Planeta C.S., Camarini R. (2020). Effects of Ethanol Exposure During Adolescence or Adulthood on Locomotor Sensitization and Dopamine Levels in the Reward System. Front. Behav. Neurosci..

[20] Quigley J.A., Logsdon M.K., Turner C.A., Gonzalez I.L., Leonardo N.B., Becker J.B. (2021). Sex differences in vulnerability to addiction. Neuropharmacology.

[21] Masur J., Boerngen R. (1980). The excitatory component of ethanol in mice: A chronic study. Pharmacol. Biochem. Behav..

[22] Camarini R., Marianno P., Rae M. (2018). Chapter three—Social Factors in Ethanol Sensitization. Int. Rev. Neurobiol..

[23] Morley-Fletcher S., Rea M., Maccari S., Laviola G. (2003). Environmental enrichment during adolescence reverses the effects of prenatal stress on play behavior and HPA axis reactivity in rats. Eur. J. Neurosci..

[24] Santos-Rocha J.B., Rae M., Teixeira A.M.A., Teixeira S.A., Munhoz C.D., Muscará M.N., Marcourakis T., Szumlinski K.K., Camarini R. (2018). Involvement of neuronal nitric oxide synthase in cross-sensitization between chronic unpredictable stress and ethanol in adolescent and adult mice. Alcohol.

[25] dos Santos J.R.B., Rae M., Teixeira S.A., Muscará M.N., Szumlinski K.K., Camarini R. (2023). The effect of MK-801 on stress-ethanol cross-sensitization is dissociable from its effects on nNOS activity. Alcohol.

[26] Neumann I.D., Krömer S.A., Toschi N., Ebner K. (2000). Brain oxytocin inhibits the (re)activity of the hypothalamo-pituitary-adrenal axis in male rats: Involvement of hypothalamic and limbic brain regions. Regul. Pept..

[27] Windle R.J., Shanks N., Lightman S.L., Ingram C.D. (1997). Central oxytocin administration reduces stress-induced corticosterone release and anxiety behavior in rats. Endocrinology.

[28] Georgiou P., Zanos P., Garcia-Carmona J.A., Hourani S., Kitchen I., Kieffer B.L., Laorden M.L., Bailey A. (2015). The oxytocin analogue carbetocin prevents priming-induced reinstatement of morphine-seeking: Involvement of dopaminergic, noradrenergic and MOPr systems. Eur. Neuropsychopharmacol..

[29] Zanos P., Georgiou P., Wright S.R., Hourani S.M., Kitchen I., Winsky-Sommerer R., Bailey A. (2014). The oxytocin analogue carbetocin prevents emotional impairment and stress-induced reinstatement of opioid-seeking in morphine-abstinent mice. Neuropsychopharmacology.

[30] Carrara-Nascimento P.F., Griffin III W.C., Pastrello D.M., Olive M.F., Camarini R. (2011). Changes in extracellular levels of glutamate in the nucleus accumbens after ethanol-induced behavioral sensitization in adolescent and adult mice. Alcohol.

[31] Didne V., van Ingelgom T., Tirelli E., Quertemont E. (2019). Long-term exposure to daily ethanol injections in DBA/2J and Swiss mice: Lessons for the interpretation of ethanol sensitization. PLoS ONE.

[32] Le A.D., Ko J., Chow S., Quan B. (1994). Alcohol consumption by C57BL/6, BALB/c, and DBA/2 mice in a limited access paradigm. Pharmacol. Biochem. Behav..

[33] Camarini R., Hodge C.W. (2004). Ethanol preexposure increases ethanol self-administration in C57BL/6J and DBA/2J mice. Pharmacol. Biochem. Behav..

[34] Crabbe J.C., Phillips T.J. (2004). Pharmacogenetic studies of alcohol self-administration and withdrawal. Psychopharmacology.

[35] Passoni I., Leonzino M., Gigliucci V., Chini B., Busnelli M. (2016). Carbetocin is a Functional Selective Gq Agonist That Does Not Promote Oxytocin Receptor Recycling After Inducing β-Arrestin-Independent Internalisation. J. Neuroendocrinol..

[36] Caligioni C.S. (2009). Assessing reproductive status/stages in mice. Curr. Protoc. Neurosci..

[37] Phillips T.J., Huson M., Gwiazdon C., Burkhart-Kasch S., Shen E.H. (1995). Effects of acute and repeated ethanol exposures on the locomotor activity of BXD recombinant inbred mice. Alcohol. Clin. Exp. Res..

[38] Legastelois R., Botia B., Naassila M. (2015). Sensitization to the stimulant motor effects of ethanol is not dependent on tolerance to ataxic or sedative properties of ethanol in female mice. Drug Alcohol. Depend..

[39] Rueda A.V.L., Teixeira A.M.A., Yonamine M., Camarini R. (2012). Environmental enrichment blocks ethanol-induced locomotor sensitization and decreases BDNF levels in the prefrontal cortex in mice. Addict. Biol..

[40] Rhodes J.S., Best K., Belknap J.K., Finn D.A., Crabbe J.C. (2005). Evaluation of a simple model of ethanol drinking to intoxication in C57BL/6J mice. Physiol. Behav..

[41] Marianno P., Abrahao K.P., Camarini R. (2017). Environmental enrichment blunts ethanol consumption after restraint stress in C57BL/6 mice. PLoS ONE.

[42] Segal D.S., Mandell A.J. (1974). Long-Term Administration of d-Amphetamine: Progressive Augmentation of Motor Activity and Stereotypy. Pharmacol. Biochem. Behav..

[43] Segal D.S., Geyer M.A., Schuckit M.A. (1981). Stimulant-induced psychosis: An evaluation of animal methods. Essays Neurochem. Neuropharmacol..

[44] Phillips T.J., Roberts A.J., Lessov C.N. (1997). Behavioral sensitization to ethanol: Genetics and the effects of stress. Pharmacol. Biochem. Behav..

[45] Richtand N. (2006). Behavioral Sensitization, Alternative Splicing, and D3 Dopamine Receptor-Mediated Inhibitory Function. Neuropsychopharmacollogy.

[46] Rae M.B., Zanos P., Georgiou P., Chivers P., Bailey A., Camarini R. (2018). Environmental enrichment enhances conditioned place preference to ethanol via an oxytocinergic-dependent mechanism in male mice. Neuropharmacology.

[47] Kuhn B.N., Kalivas P.W., Bobadilla A.C. (2019). Understanding Addiction Using Animal Models. Front. Behav. Neurosci..

[48] Robinson T.E., Berridge K.C. (1993). The neural basis of drug craving: An incentive-sensitization theory of addiction. Brain Res. Rev..

[49] Stephens M.A., Wand G. (2012). Stress and the HPA axis: Role of glucocorticoids in alcohol dependence. Alcohol Res..

[50] McGinty G., Hyland P., Shevlin M. (2019). Trauma Response and Psychosis: Investigating the Association between PTSD Symptomology and Psychotic Experiences. Eur. J. Psychotraumatology.

[51] Mitchell J.M., Arcuni P.A., Weinstein D., Woolley J.D. (2016). Intranasal Oxytocin Selectively Modulates Social Perception, Craving, and Approach Behavior in Subjects With Alcohol Use Disorder. J. Addict. Med..

[52] Greaves P. (2012). Chapter 12—Female Genital Tract. Histopathology of Preclinical Toxicity Studies.

[53] Wangikar P., Ahmed T., Vangala S., Gupta R.C. (2011). Chapter 76: Toxicology Pathology of reproductive system. Reproductive and Developmental Toxicology.

[54] Seligowski A.V., Hurly J., Mellen E., Ressler K.J., Ramikie T.S. (2020). Translational studies of estradiol and progesterone in fear and PTSD. Eur. J. Psychotraumatol..

[55] Ycaza Herrera A., Mather M. (2015). Actions and interactions of estradiol and glucocorticoids in cognition and the brain: Implications for aging women. Neurosci. Biobehav. Rev..

[56] Seale J.V., Wood S.A., Atkinson H.C., Harbuz M.S., Lightman S.L. (2004). Gonadal steroid replacement reverses gonadectomy-induced changes in the corticosterone pulse profile and stress-induced hypothalamic-pituitary-adrenal axis activity of male and female rats. J. Neuroendocrinol..

[57] King C.E., Griffin W.C., Lopez M.F., Becker H.C. (2021). Activation of hypothalamic oxytocin neurons reduces binge-like alcohol drinking through signaling at central oxytocin receptors. Neuropsychopharmacology.

[58] Rae M., Lemos Duarte M., Gomes I., Camarini R., Devi L.A. (2022). Oxytocin and vasopressin: Signalling, behavioural modulation and potential therapeutic effects. Br. J. Pharmacol..

[59] Qi J., Yang J.Y., Song M., Li Y., Wang F., Wu C.F. (2008). Inhibition by oxytocin of methamphetamine-induced hyperactivity related to dopamine turnover in the mesolimbic region in mice. Naunyn Schmiedebergs Arch. Pharmacol..

[60] Kovacs G.L., Sarnyai Z., Babarczi E., Szabo G., Telegdy G. (1990). The role of oxytocin-dopamine interactions in cocaine-induced locomotor hyperactivity. Neuropharmacology.

[61] Abrahao K.P., Ariwodola O.J., Butler T.R., Rau A.R., Skelly M.J., Carter E., Alexander N.P., McCool B.A., Souza-Formigoni M.L., Weiner J.L. (2013). Locomotor sensitization to ethanol impairs NMDA receptor-dependent synaptic plasticity in the nucleus accumbens and increases ethanol self-administration. J. Neurosci..

[62] Ribeiro A.F., Pigatto G., Goeldner F.O., Lopes J.F., de Lacerda R.B. (2008). Lack of relation between drug-seeking behavior in an addiction model and the expression of behavioral sensitization in response to ethanol challenge in mice. J. Neural Transm..

