# Supplementary material for: Carbetocin Inhibits Behavioral Sensitization to Ethanol in Male and Female Mice, Independent of Corticosterone Levels
Source: Toxics. 2023 Oct 31;11(11):893. doi: 10.3390/toxics11110893 (PMC10674331; doi:10.3390/toxics11110893)
Supplement: Supplementary file 1 [file toxics-11-00893-s001.zip › toxics-2653876-supplementary.pdf]

toxics

MDPI

Supplementary Materials:

Carbetocin Inhibits Behavioral Sensitization to Ethanol in Male and Female Mice, Independent of Corticosterone Levels

Table S1. Statistical analysis.

Behavioral Sensitization					
**Effect**	**H1 - H2 4-WAY RM ANOVA**	**D1 - D8 - D15 3-WAY RM ANOVA**		**D22 2-WAY ANOVA**	
	Male and female	Male	Female	Male	Female
pretreatment	F(1,54) = 2.2, p = 0.14	*	*	F(1,26) = 1.6, p = 0.22	F(1,28) = 2.9, p = 0.10
treatment	F(1,54) = 1.3, p = 0.25	F(1,26) = 2.3, p = 0.14	F(1,28) = 3.7, p = 0.06	*	-
pretreatment x sex	F(1,54) = 0.5, p = 0.48	-	-	-	-
treatment x sex	F(1,54) = 0.1, p = 0.79	-	-	-	-
pretreatment x treatment	F(1,54) = 1.1, p = 0.29	F(1,26) = 0.9, p = 0.33	-	*	*
pretreatment x treatment x sex	[F(1,54) = 0.1, p = 0.87	-	-	-	-
time x sex	F(1,54) = 0.2, p = 0.66	-	-	-	-
time x pretreatment	F(1,54) = 1.1, p = 0.30	*	*	-	-
time x treatment	F(1,54) = 2.5, p = 0.12	F(2,52) = 0.2, p = 0.84	F(2,56) = 0.2, p = 0.79	-	-
time x sex x pretreatment	F(1, 54) = 0.7, p = 0.42	-	-	-	-
pretreatment x	F(1,54) = 2.1, p = 0.16	F(2,52) = 0.3, p = 0.70	F(2,56) = 0.5, p = 0.58	-	-

treatment x time					
pretreatment x treatment x time x sex	F(1,54) = 2.0, p = 0.16	-	-	-	-

Corticosterone

Effect	D21			D22		
	Male and female 3-WAY ANOVA	Male 2-WAY ANOVA	Female 2-WAY ANOVA	Male and female 3-WAY ANOVA	Male 2-WAY ANOVA	Female 2-WAY ANOVA
pretreatment	F(1,48) = 0.5; p = 0.49	F(1,24)= 0.7, p = 0.39	*	*	*	*
treatment	-	F(1,24)= 1.2, p = 0.28	*	F(1,36)= 3.1, p = 0.09	*	F(1,16) = 0.1, p = 0.73
pretreatment x sex	F(1,48)= 2.2; p = 0.14	-	-	*	-	-
treatment x sex	F(1,48)= 0.1, p = 0.95	-	-	F(1,36)= 0.6, p = 0.43	-	F(1,16) = 1.2, p = 0.30
pretreatment x treatment	F(1,48)= 2.9, p = 0.09	F(1,24)= 1.1, p = 0.30	F(1,20)= 0.8, p = 0.37	F(1,36)= 0.8, p = 0.37	F(1,16)= 3.5, p = 0.08	-
pretreatment x treatment x sex	F(1,48)= 0.5, p = 0.47	-	-	-	-	-
pretreatment x cycle	-	-	F(1,20)= 0.01, p = 0.93	-	-	F(1,16)= 1.7, p = 0.21
treatment x cycle	-	-	F(1,20)= 4.1, p = 0.06	-	-	F(1,16)= 1.9, p = 0.19
pretreatment x treatment x cycle	-	-	F(1,20)= 0.03, p = 0.86	-	-	F(1,16)= 3.4, p= 0.08

BEC

Effect	Male and female 3-WAY ANOVA

treatment	F(1,32) = 0.2, p = 0.64	
pretreatment x sex	F(1,32) = 0.4, p = 0.54	
treatment x sex	F(1,32) = 0.03, p = 0.86	
pretreatment x treatment	F(1,32) = 0.4, p = 0.51	
pretreatment x treatment x sex	F(1,32) = 1.0, p = 0.32	
